# Relationship between adjustment disorder symptoms and probable diagnosis before and after second lockdown in Israel: longitudinal symptom network analysis

**DOI:** 10.1192/bjo.2022.588

**Published:** 2022-10-18

**Authors:** Yafit Levin, Rahel Bachem, Robin Goodwin, Yaira Hamama-Raz, Elazar Leshem, Menachem Ben-Ezra

**Affiliations:** School of Social Work, Ariel University, Ariel, Israel; Department of Psychology, University of Zurich, Zurich, Switzerland; Department of Psychology, University of Warwick, Coventry, UK

**Keywords:** COVID-19, adjustment disorder, longitudinal data, International Adjustment Disorder Questionnaire, statistical methodology

## Abstract

**Background:**

There is cumulative evidence of the importance of exploring the change of dynamics between symptoms over time as reflective of consolidation of psychopathology.

**Aims:**

To explore the interactions between symptoms of ICD-11 adjustment disorder before and after the second lockdown of the COVID-19 pandemic in Israel and identify the most central symptoms and their concurrent and prospective associations with probable adjustment disorder.

**Method:**

This is a population-based study drawn from a probability-based internet panel. A representative sample of the adult Israeli population was assessed at two time points (T1, pre-second lockdown, *n* = 1029, response rate 76.17%; T2, post-second lockdown, *n* = 764, response rate 74.24%). Symptoms of adjustment disorder were assessed by the International Adjustment Disorder Questionnaire (IADQ).

**Results:**

Although the overall strength of associations at the two measurement points was similar and two same communities were found, there was a significant change in their structure, with a more consolidated network at T2. The most central item was ‘difficult to relax’ in both networks. Cross-sectionally, all symptoms of failure to adapt significantly predicted adjustment disorder. ‘Worry a lot more’ (preoccupation) and ‘difficult to adapt to life’ (failure to adapt) at T1 significantly predicted this diagnosis at T2.

**Conclusions:**

Adjustment disorder symptoms consolidated during the second lockdown of the pandemic. In line with the ICD-11 conceptualisation of adjustment disorder, both preoccupation and failure-to-adapt symptoms have prognostic validity. This highlights the importance of identifying and targeting adjustment disorder symptoms during a period of stress such as the COVID-19 pandemic.

Adjustment disorder describes a maladaptive response to a psychosocial stressor and is one of the most prevalent mental health conditions worldwide.^1^ However, adjustment disorder has long been considered a controversial diagnosis owing to its vague definition in diagnostic manuals,^2,3^ with the symptom profile of the disorder only recently defined in ICD-11.^4^ ICD-11 adjustment disorder comprises two main symptom clusters: ‘preoccupation with the stressor’ (symptoms such as recurrent and distressing thoughts about the stressor or its implications) and ‘failure to adapt’ (including difficulties concentrating, sleep disturbances and an inability to recover emotionally).^4^ Symptoms must emerge within 1 month of the occurrence of the stressor and be associated with impairment in functioning.^4^ It is important to further explore the ICD-11 adjustment disorder symptoms and their development over time. Thus, the current longitudinal study's primary aim was to identify the predictive value of central symptoms of adjustment disorder for a probable diagnosis of the disorder in response to the COVID-19 pandemic.

## Diagnostic instruments

Diagnostic assessments need to be psychometrically sound, brief enough for frequent administration and clinically useful.^5,6^ To capture the symptoms of ICD-11 adjustment disorder, two self-report questionnaires have been developed. The first, the Adjustment Disorder New Module (ADNM),^7^ was instrumental in the development of the ICD-11 concept of adjustment disorder and has been used frequently in ICD-11-related research into adjustment disorder. The second, the International Adjustment Disorder Questionnaire (IADQ),^8^ corresponds more strictly to the narrative description of adjustment disorder in ICD-11. The IADQ consists of a nine-item stressor list, three items that assess preoccupation with the stressor, three items that consider symptoms of failure to adapt and three items that assess functional impairment.

To date, two studies have focused on the validation of the IADQ. The questionnaire was first validated in a nationally representative sample of adults (*n* = 1020) in Ireland.^8^ In the second validation, convenience samples were recruited in Israel (*n* = 1142) and Switzerland (*n* = 699) during the initial stages of the COVID-19 pandemic.^9^ In both studies, factorial validity was demonstrated via confirmatory factor analyses, revealing a correlated two-factor structure with an excellent fit to the data. Cronbach's alpha values were excellent for the preoccupation (α = 0.84–0.88) and failure-to-adapt items(α = 0.91–0.92) and the total IADQ score (α = 0.92–0.94). Concurrent validity was supported by strong correlations with depression, anxiety, acute stress and negative emotions, whereas correlations with post-traumatic stress disorder, complex post-traumatic stress disorder and positive emotions were weaker. Using the diagnostic algorithm of the IADQ, prevalence of probable adjustment disorder was estimated at 15.6% in Ireland,^8^ 18.8% in Switzerland and 10.2% in Israel.^9^ Initial validation efforts thus suggest that the IADQ is a valid and reliable yet parsimonious measure of ICD-11 adjustment disorder.^8,9^

## Symptom network analysis

Given the availability of standardised and validated assessment tools for ICD-11 adjustment disorder, it is now possible to investigate diagnostic criteria in greater depth. To do so a deeper understanding of the interactions among symptoms is essential. Network analysis conceptualises mental disorders as systems of connected symptoms rather than reflecting an unobservable disorder.^10^ This places the focus on understanding the individual symptoms of a syndrome, identifying those symptoms that are most central and convey high levels of clinical information.^11^ It has been argued that treatments targeting these central symptoms should be the most efficacious in alleviating psychopathology.^10^

Two recent cross-sectional studies explored symptom networks for adjustment disorder using the ADNM questionnaire. In a study including 2524 participants from Nigeria, Kenya and Ghana, preoccupation symptoms (‘I have to think about the stressful situation a lot and this is a great burden to me’) had the highest centrality.^12^ A second study, using a non-clinical sample from Switzerland, also found preoccupation symptoms were most central. In contrast, a clinical sample from the UK found failure-to-adapt symptoms to be most central. The authors suggested a temporal development, according to which adjustment disorder may be first characterised by emerging preoccupation symptoms, followed by a failure to adapt and functional impairment – the clinical manifestation of adjustment disorder.^12^ However, such cross-sectional studies could not take into account the temporal dynamics of symptoms, and thus these conclusions remained tentative.

There is increasing recognition of the importance of investigating change of dynamics between symptoms over time as reflective of consolidation of psychopathology in a network.^13^ In the current study, we used network analyses to study adjustment disorder symptoms’ dynamics before and after the second COVID-19 lockdown in Israel. A longitudinal study allows for inferences about the stability of the interaction between symptoms and the stability of the central symptoms. If a central symptom is stable over time, it maintains the same role in terms of strength in activating other symptoms. Studying such changes over time in symptom centrality or symptom relations can help elucidate the development and maintenance of the disorder. Such longitudinal data allow for testing whether an adjustment disorder diagnosis is predicted by individual central symptoms, yielding valuable information about the potential for intervention.

## Study aims and hypotheses

The COVID-19 pandemic can be viewed as a highly stressful event likely to lead to adjustment disorder.^14^ Measures for the containment of the pandemic (i.e. restrictions and lockdowns) have been practised worldwide and stressors related to lockdowns (e.g. fear of infection, social isolation, economic stressors) have been widely reported.^15,16^ Israel was one of the first countries to implement a second national lockdown as a result of a rapid infection increase (18 September to 8 November 2020). Initial analyses of the data reported in the current study showed that 28.7% had either stable-high, exacerbation or recovery trajectories of adjustment disorder.^17^ However, no study has prospectively addressed ICD-11 adjustment disorder symptom networks before and after a lockdown. The present study thus aims to: (a) identify the most central symptoms of adjustment disorder before and after the second lockdown in Israel, testing the stability of the structure and the centrality index; (b) cross-sectionally and prospectively examine associations between the most central symptoms of adjustment disorder and probable diagnosis of adjustment disorder, before and after the second lockdown.

We assumed that the structure of ICD-11 adjustment disorder would be confirmed and that, as often occurs in psychopathology, the networks would be more consolidated in the second measurement. Based on the limited number of previous studies, we hypothesised that the preoccupation cluster would be most central as the sample is non-clinical. We hypothesised that in both measurements, the central symptom would be stable. Finally, the most central symptom would predict the diagnosis, both concurrently and prospectively.

## Method

### Recruitment and eligibility

Data were collected from 3 August to 30 August 2020 (Time 1, T1) and 15 November to 3 December (Time 2, T2). Eligibility criteria specified that participants should be aged 18 or older, Israeli residents at the time of the study, able to give informed consent and fluent in Hebrew.

### Sample size

Power analysis and sample size guidelines for network analysis are as yet unclear; some have suggested that at least three individuals per parameter are necessary,^18,19^ whereas others require at least five participants per node in the network.^20^ The current study included data from 764 participants for two networks with a total of 12 nodes (63 participants per node). This indicates high power for the network analysis. For logistic regressions, we estimated that a minimum of 320 participants would be required to detect effect sizes of 0.10, with 90% power and a 5% significance level, based on the inclusion of 11 explanatory variables (5 background variables and 6 individual symptoms): our data also exceeded the minimum sample size of 500 suggested in a logistic regression simulation study.^21^

### Sampling and procedures

The study was conducted according to the STROBE guidelines for observational studies. We used Israel's iPanel company to deploy the COVID-19 Mental Health Survey among participants aged 18–71. This panel is a probability-based panel with over 100 000 members.^22^ The panel consists of adults aged 18–85 who have given their consent to be contacted about surveys. Panel recruitment is dynamic and constant using a range of online methods. iPanel adheres to the stringent standards of ESOMAR, an international association for market, social and opinion researchers.

A quota sampling approach was used, with quotas meeting the Israeli national census data on age and gender, as specified by the Israeli Bureau of Statistics census data. The final data-set was weighted according to factors of age and gender to represent the internet-using population of adults between 18 and 71 years of age living in Israel.

The authors assert that all procedures contributing to this work comply with the ethical standards of the relevant national and institutional committees on human experimentation and with the Helsinki Declaration of 1975, as revised in 2008. All procedures involving human patients were approved by Ariel University's Institutional Review Board (AU-SOC-YHR-20200616).

The sample was administered online, and all participants signed an electronic informed consent form. At T1, out of 1351 invitations sent, 1029 responded (response rate 76.17%); at T2, out of 1029 participants in T1, 764 responded (response rate 74.25%). We conducted a set of sensitivity analyses at T1 comparing those who did answer the survey with those who did not (*n* = 322) on the following key demographic factors: age (*t*(1049) = 1.10, *P* = 0.271), gender (χ²(1) = 2.65, *P* = 0.104), marital status (χ²(4) = 1.33, *P* = 0.856), income (χ²(4) = 2.77, *P* = 0.594) and education (χ²(5) = 6.84, *P* = 0.145). No significant differences were found between the groups. Demographic variables assessed included age (mean 40.75 years; s.d. = 14.75; range 18–71), gender (50.5% of the sample were women) and relationship status (58.3% of the sample were in a committed relationship).

### Measurements

#### Demographics

Demographic variables assessed included age, gender, relationship status, education and income level (five-point Likert scale ranging from 1, much below average, to 5, much above average). The risk group for COVID-19 was assessed using criteria from the World Health Organization (WHO) and US Centers for Disease Control: ‘Do you suffer from one of the following medical conditions: hypertension, diabetes, cardiovascular disease, chronic respiratory disease, chronic obstructive pulmonary disease and cancer’. For further details see supplementary Table 1 available at https://doi.org/10.1192/bjo.2022.588.

#### Adjustment disorder

Adjustment disorder according to ICD-11 was measured using the 9-item International Adjustment Disorder Questionnaire (IADQ).^8^ The IADQ assesses core symptoms of the disorder (six items for preoccupation and failure to adapt; three items for functional impairment) on a Likert scale (0, not at all, to 4, extremely). In this study, the IADQ symptoms referred to the specific stressor of the COVID-19-related lockdown. A tenth question assesses the duration of symptoms. The algorithm for a probable diagnosis of ICD-11 adjustment disorder requires the presence of a psychosocial stressor, at least one preoccupation symptom rated ≥2, at least one failure-to-adapt symptom rated ≥2 and evidence of functional impairment rated ≥2. Reliabilities were high (T1, α = 0.93; T2, α = 0.94).

### Statistical method

#### Regularised partial correlation networks

We estimated Gaussian graphical models (GGMs) for partial pairwise association parameters between all nodes. In the GGMs, edges can be understood as conditional dependence relations among symptoms. Symptoms that are not connected are conditionally independent. With 6 symptom nodes, 15 pairwise association parameters are estimated. We controlled for false positives by using the graphical least absolute shrinkage and selection operator (graphical LASSO),^23^ which sets very small edges to zero (implemented in the R-package qgraph for Windows, version 1.9.2^24^). This procedure employs a regularisation technique that conservatively identifies only relevant edges in order to uncover underlying network structure.^25^ This visualises sparse networks using part correlations and considers the ordinal scale of the questionnaire;^18^ further information regarding network estimation and stability and accuracy of both edges and centrality can be found in the supplementary materials.

#### Community detection

The spinglass algorithm was used to identify communities of items in the network. This method divides networks into groups, so that each community contains several densely connected clusters of nodes.

#### Bridge symptoms

We used the *bridge* function of the networktools package (R for Windows)^26^ to identify bridge symptoms between the communities in each network. Bridge strength is defined as the sum of the absolute values of all edges that exist between a node and all nodes that are not in the same cluster. Examination of the bridge symptom between the subsets revealed in a community analysis is essential to the understanding of the underlying mechanisms in the overall network.

#### Network stability

We examined the stability of the individually estimated networks, estimating 95% confidence intervals around edge weights and a correlation-stability coefficient for strength centrality. More information regarding the network analysis techniques can be found in the supplementary materials (data analysis section) and in the tutorial by Epskamp et al.^19^

#### Network comparisons

To compare differences between networks, we estimated network differences between each pair of networks using the Network Comparison Test (NCT) package in R for Windows.^27^ See further information on network comparison techniques in the supplementary materials. The use of symptom networks in two measurements is acknowledged in psychopathology.^28^

#### Logistic regressions

Three logistic regressions were conducted to predict probable diagnosis of adjustment disorder by individual symptoms. The first two regressions were cross-sectional, for both T1 and T2. The third regression prospectively predicted probable diagnosis at T2 by individual symptoms of adjustment disorder at T1. In step 1, age, gender, education, self-rated health and risk group membership for COVID-19 were included in the model. In the second step, we added individual symptoms of ICD-11 adjustment disorder. We tested whether the contribution of the central symptoms would concurrently and prospectively significantly contribute to probable diagnosis of adjustment disorder, considering both specific central symptoms and the subscale they represent.

## Results

### Descriptive information

Table 1 shows the mean scores on the six IADQ core symptom items across the two measurements. Rates of probable adjustment disorder were high in both measurements: 20.7% at T1 and 19.8% at T2.

**Table 1:** Mean scores and endorsement rates (≤2) for the nine items on the International Adjustment Disorder Questionnaire at time points T1 and T2 (*n* = 764)

	T1		T2	
	Mean (s.d.)	*n* (%)	Mean (s.d.)	*n* (%)
**Preoccupation**				
1 I worry a lot more since the stressful event(s)	1.69 (1.26)	403 (52.8%)	1.44 (1.12)	331 (43.3%)
2 I can't stop thinking about the stressful event(s)	1.30 (1.22)	298 (39%)	1.12 (1.10)	264 (34.5%)
3 I often feel afraid about what might happen in the future since the stressful event(s)	1.58 (1.34)	359 (47%)	1.37 (1.17)	316 (41.3%)
**Failure to adapt**				
4 I find it difficult to adapt to life since the stressful event(s)	1.17 (1.25)	262 (34.3%)	1.00 (1.06)	219 (28.7%)
5 I find it difficult to relax and feel calm since the stressful event(s)	1.09 (1.24)	243 (31.8%)	0.94 (1.07)	211 (27.6%)
6 I find it difficult to achieve a state of inner peace since the stressful event(s)	1.04 (1.23)	235 (30.8%)	0.95 (1.10)	217 (28.4%)
**Total scale score**		158 (20.7%)		151 (19.8%)
**Symptom time criterion** (1 month of the stressful event(s))		408 (53.4%)		373 (48.8%)
**Functional impairment**				
7 Affected your relationships or social life?	1.16 (1.28)	243 (31.8%)	1.04 (1.16)	230 (30.1%)
8 Affected your ability to work or your educational life?	1.12 (1.32)	227 (29.7%)	1.03 (1.21)	234 (30.7%)
9 Affected any other important part of your life?	1.30 (1.31)	286 (37.4%)	1.15 (1.18)	268 (35.0%)

### Regularised partial correlation networks across the three samples

#### Network estimation

**Fig. 1:**
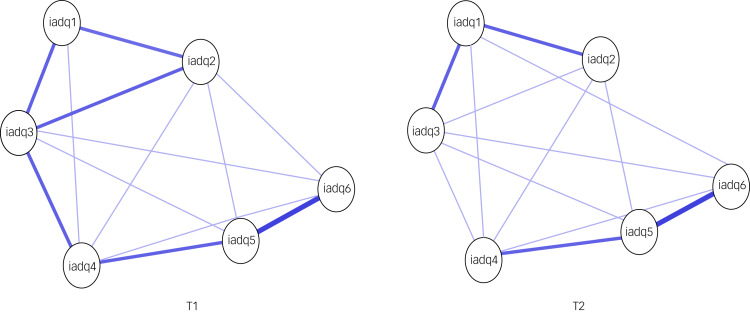
Networks of adjustment disorder symptoms over time using average spring layout. Nodes represent items on the International Adjustment Disorder Questionnaire and edges show regularised partial correlations with least absolute shrinkage and selection operator (LASSO) penalty. Distances between nodes and thickness of edges relate to the size of their partial correlations. Blue edges indicate positive relations. iadq1–iadq6 denote items on the International Adjustment Disorder Questionnaire: 1 I worry a lot more; 2 I can't stop thinking; 3 I often feel afraid about what might happen in the future; 4 I find it difficult to adapt to life; 5 I find it difficult to relax and feel calm; 6 I find it difficult to achieve a state of inner peace.

Estimated networks are shown in supplementary Fig. 1. To enhance visual comparability of edges, we estimated the average layout of the three networks, presenting networks using this layout in Fig. 1. In the T1 network, 14 of 15 possible edges were non-zero; 15 of 15 possible edges were non-zero at T2, demonstrating high connectivity of symptoms in both networks.

#### Network estimation using community analysis

Consistent communities were revealed across networks in 100% of the extractions, which supported a two-community interpretation, and verified the two dimensions of preoccupation (IADQ items 1–3) and failure to adapt (items 4–6).

### Network comparisons

Network comparison tests showed that global strength values per group were 2.77 and 2.80 for T1 and T2 respectively (S statistic 0.031, *P* = 0.41). The T1 network structure differed significantly from the T2 structure (mean 0.19, *P* = 0.05): the level of overall connectivity was similar, but the pattern of associations differed.

#### Bridge strength

For the T1 network, the communities were more distinct (connections between them were weak) compared with T2 (estimates of bridge strength are presented in supplementary Fig. 6). Item 3 had the highest bridge strength in both networks, but the bridge symptoms in the T2 network had greater strength compared with the bridge strength in T1. In T1, items 2 and 3 showed the highest bridge strength. However, in T2, the communities were more interrelated, whereby items 2–5 had the highest strength centrality.

#### The inter-community interrelations

Visual inspection of the networks showed that the inter-community correlations between symptoms were strong across measurements, with the most significantly robust connection in both networks between the failure-to-adapt items ‘difficult to relax’ (item 5) and ‘difficult to achieve a state of inner peace’ (item 6). In the T1 network, the next strongest connections were between the failure-to-adapt items ‘difficult to adapt’ (item 4) and ‘difficult to relax’ (item 5) on the failure-to-adapt subscale; and between the preoccupation items ‘cannot stop thinking’ (item 2) and ‘worry a lot more’ (item 1) and ‘cannot stop thinking’ (item 2) and ‘afraid about what might happen in the future’ (item 3).

In the T2 network, the next strongest connection was between the preoccupation items ‘worry a lot more’ (item 1) and ‘afraid about what might happen in the future’ (item 3). Then, the next strong items were between the preoccupation items ‘worry a lot more’ (item 1) and ‘cannot stop thinking’ (item 2) and between the failure-to-adapt items ‘difficult to adapt’ (item 4) and ‘difficult to relax’ (item 5).

### Network stability

To confirm the visual similarity of networks, we used Spearman correlations of edge weights for all combinations of networks (see supplementary materials). Accuracy of the edges and of the centrality strength index were large and satisfactory (supplementary material ‘Network stability’ and supplementary Figs 2–5).

### Network inference

Standardised strength centrality estimates are presented in supplementary Fig. 7. Item 5 (‘difficult to relax’) had the highest strength centrality in both networks. Nodes with the smallest centrality differed between networks, although in both networks these were from the preoccupation subscale (T1 network: ‘worry a lot more’ (item 1); T2: ‘cannot stop thinking’ (item 2).

### Predicting probable diagnosis of adjustment disorder

As illustrated in Table 2, cross-sectional analyses showed that none of the background variables contributed to probable diagnosis of adjustment disorder. The three failure-to-adapt symptoms were associated with an increased ratio to probable diagnosis of adjustment disorder: ‘I find it difficult to adapt to life’ (item 4: OR = 1.80 and 1.60 at T1 and T2 respectively); ‘I find it difficult to relax and feel calm’ (item 5: OR = 1.60 and 1.71 at T1 and T2 respectively); and ‘I find it difficult to achieve a state of inner peace’ (item 6: OR = 1.48 and 2.35 at T1 and T2 respectively). None of the preoccupation symptoms were significantly associated with adjustment disorder diagnosis.

**Table 2:** Logistic regression of factors predicting probable adjustment disorder diagnosis at time points T1 (left side of /) and T2 (right side of /)

	*B*	s.e.	Wald	*P*	OR
Age	−0.01/0.00	0.01/0.01	2.78/0.00	0.096/0.981	0.98/1.00
Gender (reference group: men)	0.31/0.10	0.25/0.26	1.55/0.16	0.213/0.693	1.37/1.11
Education	0.11/−0.05	0.10/0.11	1.17/0.24	0.280/0.626	1.12/0.95
Self-rated health	0.30/0.08	0.21/0.22	2.05/0.14	0.152/0.712	1.35/1.08
Being in risk group for COVID-19 (reference group: not at risk)	0.24/−0.40	0.31/0.32	0.61/1.54	0.435/0.215	1.27/0.673
**Individual symptoms of adjustment disorder**					
Preoccupation (items 1–3)					
1 I worry a lot more	0.24/0.26	0.14/0.18	2.92/2.02	0.087/0.155	1.28/1.29
2 I can't stop thinking	0.15/–0.07	0.18/0.19	0.65/0.13	0.421/0.720	1.16/0.933
3 I often feel afraid about what might happen in the future	0.10/0.14	0.16/0.19	0.36/0.52	0.546/0.469	1.10/1.145
Failure to adapt (items 4–6)					
4 I find it difficult to adapt to life	0.57***/0.47**	0.16/0.19	13.49/6.45	0.000/0.010	1.80/1.60
5 I find it difficult to relax and feel calm	0.47**/0.54*	0.20/0.23	5.56/5.37	0.010/0.021	1.60/1.71
6 I find it difficult to achieve a state of inner peace	0.39*/0.85***	0.18/0.20	4.91/17.94	0.027/0.000	1.48/2.35

* *P* < 0.05, **P < 0.01, ***P < 0.001.

In the prospective logistic regression (Table 3), two individual symptoms at T1 predicted the probable diagnosis of adjustment disorder at T2: ‘I worry a lot more since the stressful event’ (item 1, preoccupation: OR = 1.277) and ‘I find it difficult to adapt to life since the stressful event’ (item 4, failure to adapt: OR = 1.50).

**Table 3:** Logistic regression of factors predicting probable adjustment disorder diagnosis at time point T2 by individual symptoms at T1

	*b*	s.e.	Wald	*P*	OR
Age	−0.01	0.01	3.33	0.068	0.986
Gender (reference group: men)	0.37	0.22	2.87	0.090	1.444
Education	−0.02	0.09	0.07	0.794	0.978
Self-rated health	0.25	0.18	2.07	0.151	1.287
Being in risk group for COVID-19 (reference group: not at risk)	−0.50	0.26	3.89	0.050	0.605
**Individual symptoms of adjustment disorder**					
T1 Preoccupation (items 1–3)					
1 I worry a lot more	0.24*	0.12	3.99	0.046	1.28
2 I can't stop thinking	−0.03	0.16	0.03	0.866	0.97
3 I often feel afraid about what might happen in the future	0.02	0.14	0.01	0.908	1.02
**T1 Failure to adapt (items 4–6)**					
4 I find it difficult to adapt to life	0.41**	0.13	9.44	0.002	1.50
5 I find it difficult to relax and feel calm	0.14	0.17	0.73	0.393	1.16
6 I find it difficult to achieve a state of inner peace	0.19	0.16	1.49	0.223	1.21

* *P* < 0.05, **P < 0.01.

## Discussion

This is the first study that estimates the network of ICD-11 adjustment disorder symptoms at two time points. This longitudinal assessment allows for examination of network stability over time and for evaluation of the predictive role of individual symptoms and the probable diagnosis of ICD-11 adjustment disorder. Although the overall strength of associations at the two measurement points was similar, there was a significant change in structure, manifested in a more consolidated structure at T2. Two failure-to-adapt symptoms (‘difficult to relax’ and ‘difficult to achieve a sense of inner piece’) were most strongly connected in both networks, with ‘difficult to relax’ the most central symptom in both measurements. Cross-sectionally, all symptoms of failure to adapt significantly predicted the diagnosis. Prospectively, ‘worry a lot more’ (preoccupation) and ‘difficult to adapt to life’ (failure to adapt) at T1 significantly predicted diagnosis at T2.

### Network structure

In both networks, we found two communities representing preoccupation and failure to adapt. Strong associations were observed between individual preoccupation symptoms and between failure-to-adapt symptoms, but weaker associations between these core symptom clusters. This confirms our hypothesis and replicates earlier network studies in non-clinical^12^ and clinical samples^29^ and confirms the structure of adjustment disorder suggested in ICD-11. We report the first study to show that overall connectivity of adjustment disorder symptoms remained equally strong over 3 months, further confirming the validity of the adjustment disorder symptoms in ICD-11.

Despite the stability of the overall strength of associations at the two measurement points, there was a significant change in the structure and invariance between networks. This manifested, as hypothesised, in a more consolidated structure at T2, in which preoccupation items and failure-to-adapt items were more interconnected than at T1, when the associations within core symptom clusters were weaker. This manifested in higher strength of the bridge symptoms in the T2 network compared with T1. Connectivity (i.e. the strength of the associations that exist between symptoms) may be informative for the study of prognosis and treatment response:^30^ individuals with strongly associated symptom networks (with higher bridge strength and more bridging symptoms) may be less responsive to treatment, regardless of the overall severity of symptoms. The current results suggest that adjustment disorder may have ‘toughened’ over time, making it more difficult to treat. This emphasises the importance of prevention strategies and timely intervention in adjustment disorder.

The two failure-to-adapt symptoms ‘difficult to relax’ and ‘difficult to achieve a sense of inner piece’ were most strongly connected in both networks, and ‘difficult to relax’ was also the most central symptom in both measurements. It may be argued that these constructs are similar, as achieving ‘inner peace’ entails relaxation of both the physical body and the mind.^31^ Problems with relaxation relate to the body's physical ‘fight or flight’ reaction to perceived threat, such as the COVID-19 pandemic. Stress response syndromes such as adjustment disorder can be treated by either reducing the threat reaction or increasing the relaxation response,^32^ with relaxation techniques combined with psychoeducation useful for treating adjustment disorder.^33^ Yoga meditation techniques^34^ and autogenic training^35^ are effective in reducing symptoms of adjustment disorder. Such interventions may also be useful for the management of COVID-19-related adjustment disorder symptoms.

Contrary to our hypothesis, failure to adapt was observed as most central at both time points. This is surprising as previous studies suggested that the most central symptom of adjustment disorder in non-clinical samples typically belongs to the preoccupation factor.^12^ However, this previous work used the ADNM questionnaire to assess adjustment disorder symptoms, which includes items such as concentration difficulties that may belong to both preoccupation and failure to adapt. More research is needed to establish whether findings derived from using these different questionnaires overlap. We also note that the current study took place at the beginning of the COVID-19 pandemic, when the threat was perceived most acutely and rates of adjustment disorder were high. This may result in our present sample resembling a clinical rather than a non-clinical sample.

### Predicting probable diagnosis

In our cross-sectional analysis, all symptoms of failure to adapt significantly predicted probable diagnosis, but none of the preoccupation symptoms did. This confirms our hypothesis that the most central symptom would predict the diagnosis and our expectation that preoccupation symptoms provide the grounds for the development of adjustment disorder in non-clinical samples, whereas failure-to-adapt symptoms have a greater significance in the clinical context.^29^ However, longitudinal research with clinical samples is needed to clarify the mechanisms underlying the development of the disorder.

In the longitudinal analysis, items from both core symptom clusters predicted probable diagnosis. The preoccupation item ‘I worry a lot more’ and the failure-to-adapt item ‘I find it difficult to adapt to life’ at T1 were both predictors of diagnosis at T2. Difficulties in adapting to life could represent a functional impairment, which may explain its prognostic value. Similarly, ‘I worry a lot more’ represents preoccupation in terms of both cognitive and emotional burden. These aspects are both found in the remaining preoccupation items ‘I can't stop thinking’ and ‘I often feel afraid’. In summary, our prospective analysis points out that both core symptom clusters have clinical value in the process of disorder consolidation and supports the two-symptom-cluster conceptualisation of adjustment disorder in ICD-11.

### Strengths and limitations

This study has both strengths and limitations. We benefitted from employing a quota-based representative Israeli sample and from the use of two time points and novel statistics to compare network strength, centrality and connectivity. A potential bias in the study is the use of self-report questionnaires. Although we report on a large sample with good response rates (74.24%), we cannot be sure whether attrition led to specific bias among variables not observed in the study. Moreover, although we used longitudinal data, we did not include auto-regressive associations. This indicates that we cannot infer causality; future longitudinal (within-person) network research is essential to provide information on the stability of symptoms over time. Finally, we assessed adjustment disorder symptoms related to the Israeli lockdown during the COVID-19 pandemic. The pandemic represents an unprecedented large-scale event affecting populations worldwide and thus must be considered a unique stressor. Future research should explore whether the results replicate among individuals exposed to other stressors, particularly personal events such as severe physical illness, job loss or divorce.

### Clinical implications

The present findings show that adjustment disorder symptoms consolidated during the second lockdown of the COVID-19 pandemic. From a clinical perspective, our data therefore emphasise the importance of the early identification and targeting of adjustment disorder symptoms during a stressful mass event such as the COVID-19 pandemic. This could be achieved by primary healthcare physicians including adjustment disorder screening as part of patient visits. A stepped care approach may be the most realistic option for providing support to large numbers of persons showing adjustment difficulties. During a pandemic, where in-person consultation is limited, this might include self-help interventions delivered online^36,37^ and text-based self-help interventions (bibliotherapy).^38^

## Data Availability

All data relevant to the study are included in the article or are available from the corresponding author on request.

## Supplementary material

For supplementary material accompanying this paper visit http://doi.org/10.1192/bjo.2022.588. click here to view supplementary material
