# Supplementary material for: Relationship between adjustment disorder symptoms and probable diagnosis before and after second lockdown in Israel: longitudinal symptom network analysis
Source: BJPsych Open. 2022 Oct 18;8(6):e186. doi: 10.1192/bjo.2022.588 (PMC9634604; doi:10.1192/bjo.2022.588)
Supplement: Supplementary file 1 [file S2056472422005889sup001.doc]

**Demographics**

Table SM1 shows descriptives statistics for the background variables in the two measurements.

**Data analysis**

This section provides more detailed information about the statistical procedures and the used software. Network stability and accuracy were measured by methods previously described in detail (1). We used R-package *bootnet* to establish robustness (2).

**Network estimation**

The symptom network was estimated for all symptoms using the R-package qgraph (3). Networks were estimated using regularized partial correlation models in the R-package qgraph that present the unique, independent relationships between symptoms (1). The network is weighted and undirected due to the cross-sectional nature of the study. Questionnaire data of AjD symptoms were answered on an ordinal scale; thus, we estimated a matrix of polychoric correlations. The networks are presented in figure SM1.

**Visualization with the Graphical Lasso** This method directly estimates the inverse of the covariance matrix (4). It shrinks small edges and many parameters to zero by estimating a penalized [maximum likelihood](https://www-sciencedirect-com.ezprimo1.idc.ac.il/topics/medicine-and-dentistry/maximum-likelihood-method) solution based on the Extended [Bayesian Information Criterion](https://www-sciencedirect-com.ezprimo1.idc.ac.il/topics/medicine-and-dentistry/bayesian-information-criterion) (EBIC) (5). For ease of visual comparison, the networks were restricted to a consistent “average layout”, presented across samples.

**Network stability**

There are no clear boundaries to interpret the results of the stability analyses. The confidence intervals around the edge weights were moderately large (Figure SM2). The generally narrow bootstrapped CIs imply that interpreting the order of most edges in the network can be done with relatively high confidence. About one third of the nonzero edges had 95%-CIs that marginally included zero.

We performed two procedures to check the accuracy and stability of the results (the suggested threshold for strong stability is 0.5: (A) the accuracy of estimated edge-weights (figure SM2), (B) the stability of centrality indices (figure SM3) after subsetting the data (6). The accuracy of the estimated edge-weights was satisfactory for both measurements (T1 0.75 CI 95% 0.672, 1.00, and T2 0.75 CI 95% 0.673, 1.00). The correlation stability coefficient for the strength centrality metric was satisfactory for T1 (.672 CI 95% 0.595, 0.750) and T2 (0.673 CI 95% .594, .750), which shows that the centrality strength indexes were stable under subsetting cases in both measurements. Figure SM4 displays the edges that significantly differ from each other and Figure SM5 displays the centrality estimates of all six items that significantly differ from each other.

**Network Comparisons**

Overall connectivity can be summarized by global strength and is defined as the weighted absolute sum of all edges in the network. The distance S, based on global strength between two networks is presented. The Invariant Network Structure concerns the structure of the network as a whole. The test of network structure invariance evaluates the observed value of M in the data against the reference distribution of M that arises from a random permutation of group membership across cases (7).

**Community detection.** The *spinglass* algorithm was used to identify communities of items in the item network. It is based on the principle that the edges should connect nodes of the same community, whereas nodes belonging to different communities should not be connected (8) *spinglass* algorithm was applied to the network using the *igraph R*-package. Since this algorithm can yield different results in the same sample, we run the algorithm 1000 times with a different random seed for each run, and extracted the number of communities with the highest frequency. Interested readers are referred to Eiko Fried’s tutorial on this topic: <http://psych-networks.com/r-tutorial-identify-communities-items-networks/>).

**Bridge node analysis.** To add to previous community findings, we perform bridge node analysis (9) on the models. Figure SM6 presents the bridge strength of the nodes of the T1 and T2 networks. For this purpose, adjusted measures are calculated that take into account the membership of the items in their respective communities. Only connections to items of the other community are considered in the procedure.

**Centrality index:** Standardized strength centrality estimates are presented in Fig. SM7 in supplementary materials. Item 5 ('difficult to relax') had the highest strength centrality in both networks. Nodes with the smallest centrality differed between networks, although in both networks these were from the 'preoccupation' subscale (T1 network: 'worry a lot more' (item 1); T2 'cannot stop thinking' (item 2).

Table SM1. Participant demographics and Israeli population values

|  | **Participants (n = 1029)** | **Israel population (N = 9,291,000)** |
| --- | --- | --- |
|  | n (%) | n (%) |
| **Sex** |  |  |
| **Male** | 509 (49.5 %) | 49.7% |
| **Female** | 520 (50.5%) | 50.3% |
| **Age groups (years)*** |  |  |
| **18-22** | 180 (13.3%) | 10.1% |
| **23-29** | 218 (16.1%) | 15.9% |
| **30-39** | 291 (21.5%) | 24% |
| **40-49** | 240 (17.8%) | 20% |
| **50+** | 422 (31.2%) | 30% |
| **Education** |  |  |
| **Elementary school** | 9 (.7%) | 1.9% |
| **High school no diploma** | 132 (9.2%) | 8% |
| **Graduate high school with diploma** | 312 (23.1%) | 22% (**Graduate high school/ with diploma** **42%)** |
| **higher education with no diploma** | 292 (21.6%) | 17% |
| **undergraduate diploma** | 386 (28.6%) | 20% **(Higher diploma - academic/not academic 50.9%)** |
| **post graduate diploma** | 220 (16.3%) | 11% |
| **Income** |  | **Mean income 13,558 NIS (2,570 GBP)** |
| **much below average** | 281 (21.1%) | 26.9% |
| **a little below average** | 237 (17.8%) | N/A |
| **about average** | 332 (24.9%) | 34.1% (based on incomes from all resources to a household) |
| **a little above average** | 355 (26.7%) | N/A **above average – 28%** |
| **much above average** | 127 (9.5%) | N/A |
| **Marital Status** |  |  |
| **Single** | 431 (31.9%) | 30% |
| **Married** | 796 (58.9%) | 61% |
| **Divorced** | 107 (7.9%) | 6% |
| **Separated** | 9 (.7%) | 1% |
| **Widowed** | 8 (.6%) | 2% |
| **COVID-19 Risk Group according to the WHO criteria.** |  |  |
| **Yes** | 240 (23.3%) | N/A |
| **No** | 789 (76.7%) | N/A |

Notes. Israel population estimates from Office for National Statistics, end year estimates 2018.


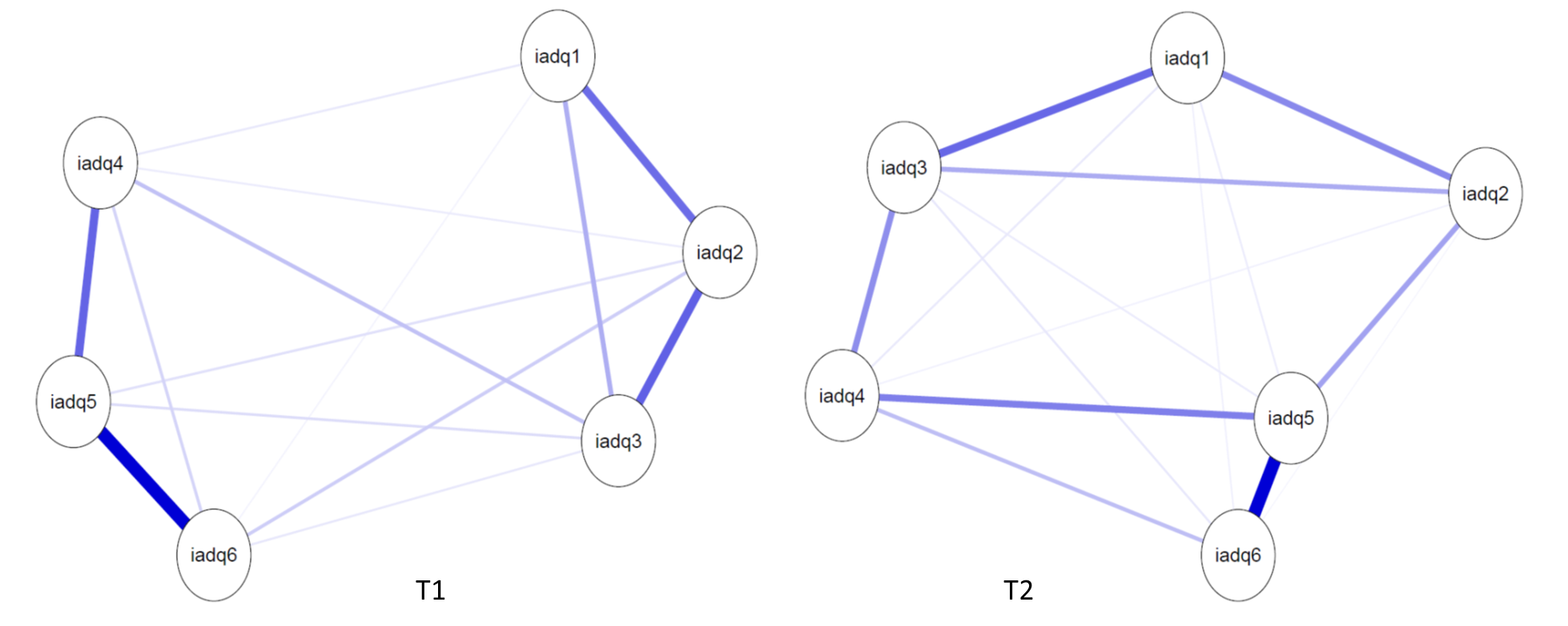


**Fig SM1. Networks of Adjustment Disorder symptoms over time**. Nodes represent Adjustment Disorder items and edges Regularized partial correlations with LASSO penalty. Distances among nodes and thickness of edges relate to size of their partial correlations. Blue edges indicate positive relations. The full items can be found in table 2.


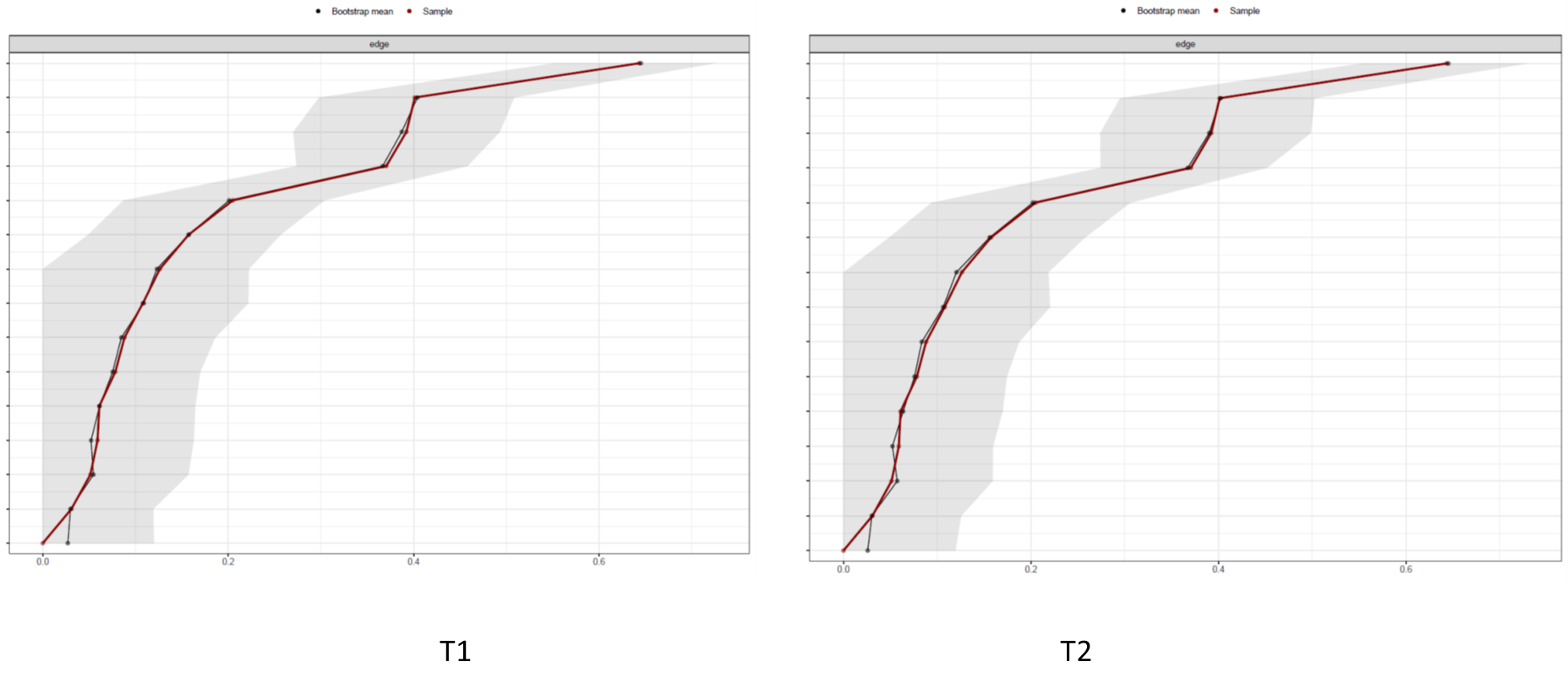


**Fig SM2.** Stability analysis -- accuracy of edge weights. Red lines are Point estimates and grey are the 95% bootstrap confidence intervals of network edges (based on partial correlations between items) for the three estimated networks.


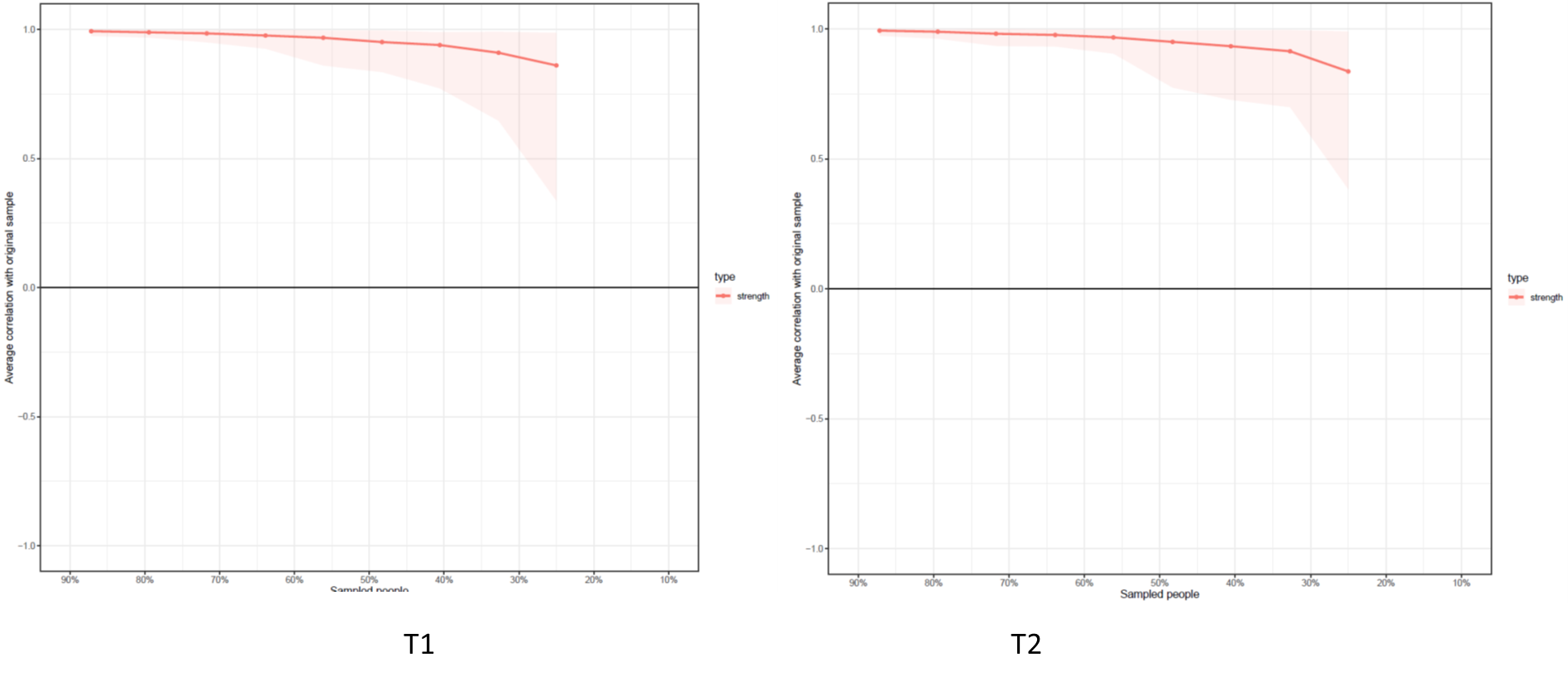


**Figure SM3. Stability Analysis**. Centrality bootstrap. Correlation of the original strength centrality order with the order of strength centrality in subsets of the data. The correlation after dropping a substantial number of participants is high for the centrality metric strength, which means that this centrality estimate can be considered stable in all three samples.


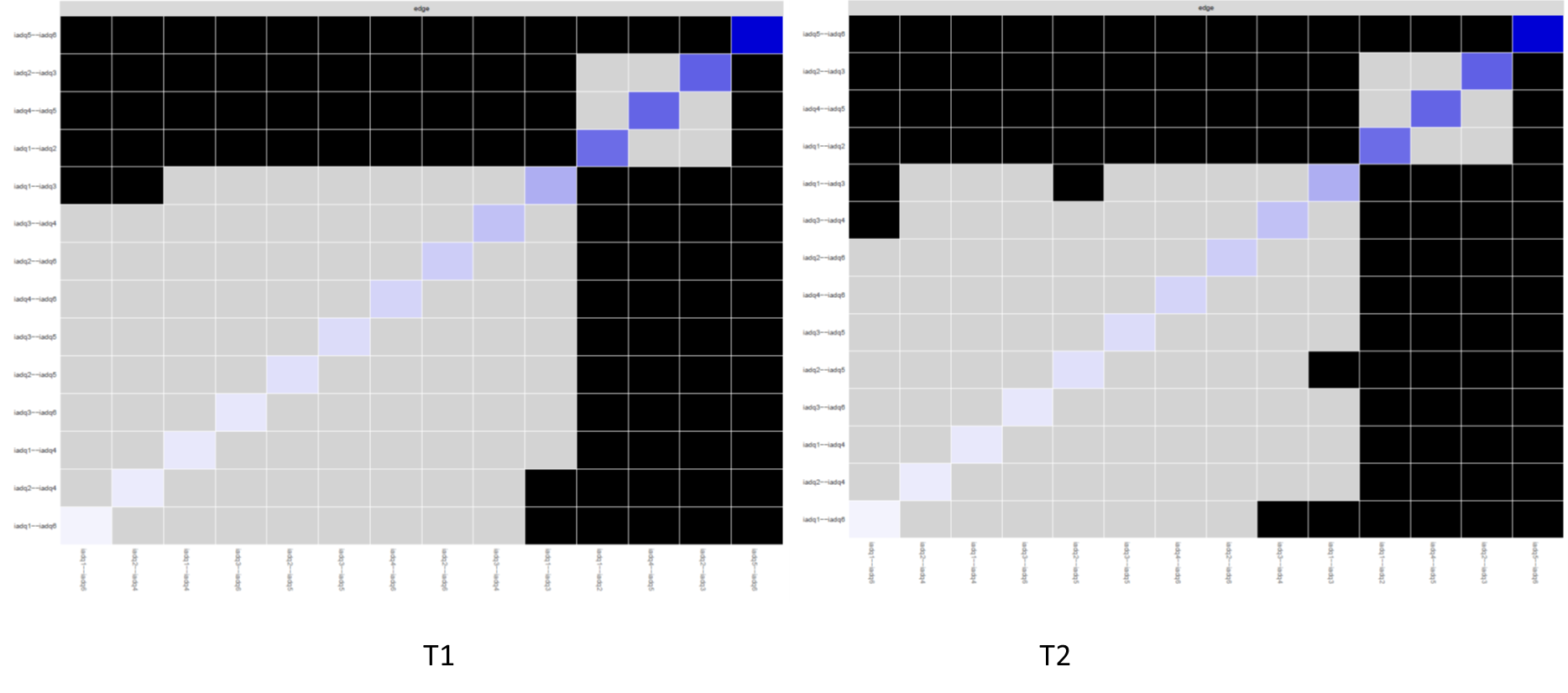


**Figure SM4. Edge weights difference test.** Black boxes represent significant differences between edge weights. The test does presently not correct for multiple testing.


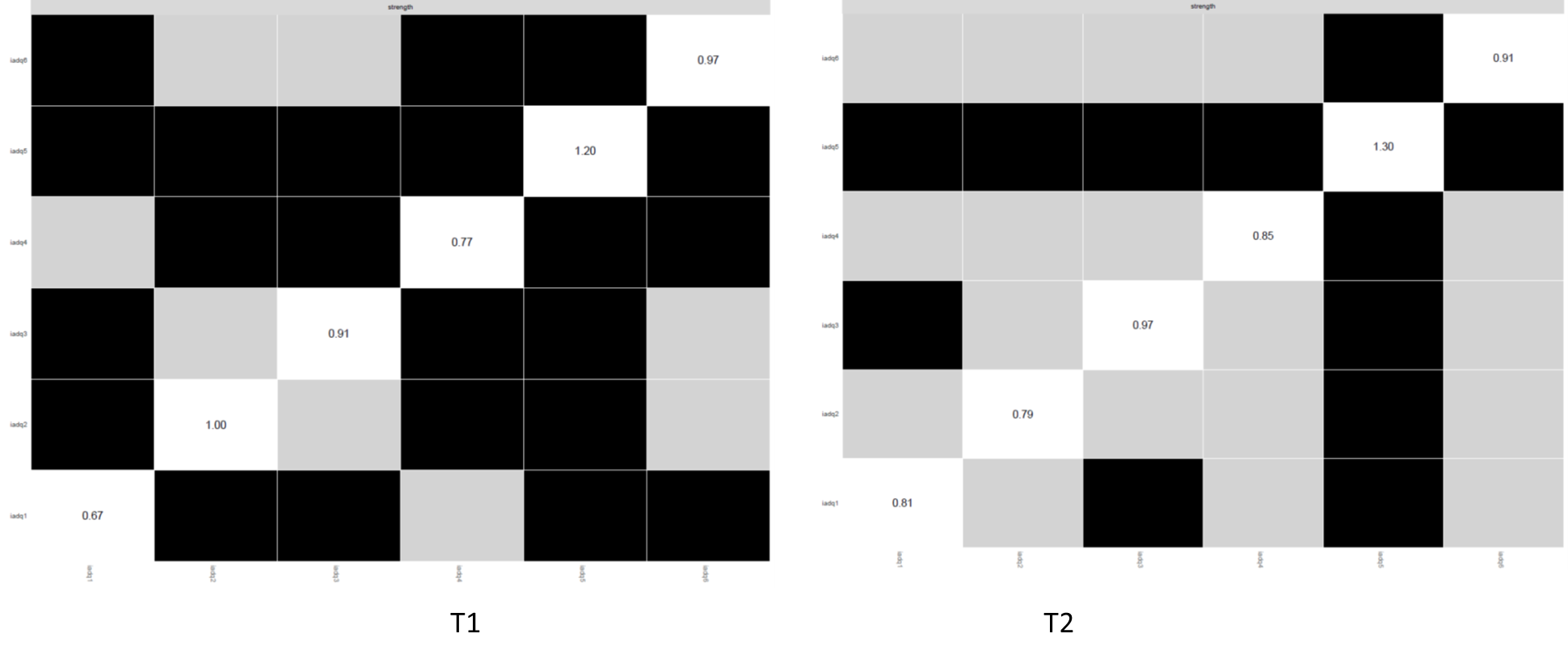


**Figure SM5. Centrality difference test.** Standardized strength centrality values are shown in the diagonal, black boxes represent significant differences in centrality estimates. The test does presently not correct for multiple testing.

**
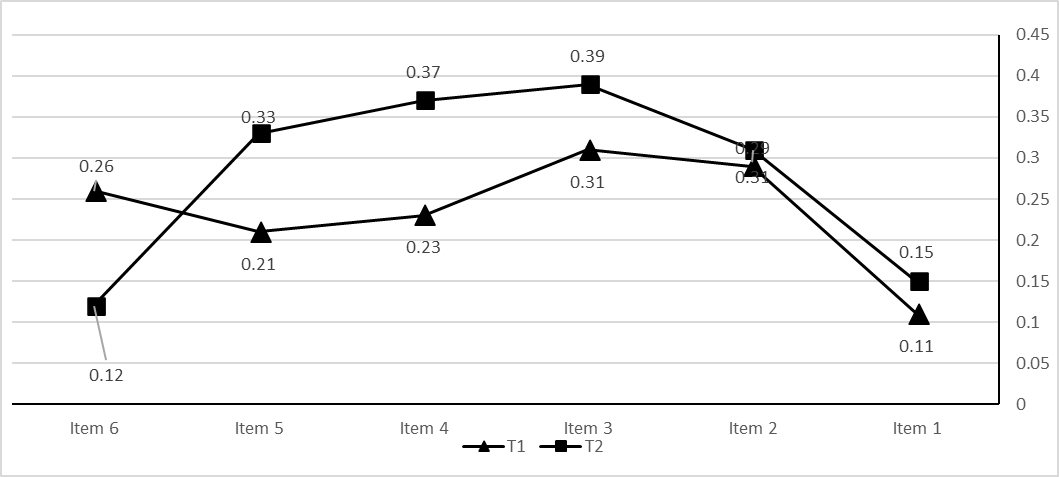
**

**Fig SM6. Node Bridge Strength centrality for the sample networks of Adjustment Disorder symptoms over time**. The full items can be found in table 2.


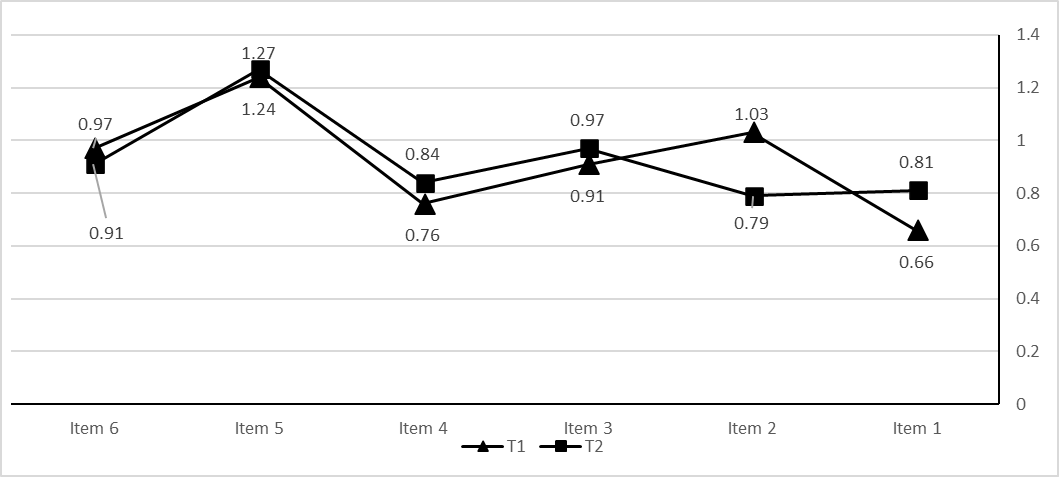


**Fig SM7.** **Standardised node strength centrality for the sample networks**. The full items can be found in Table 1.

.

**References**

1. Knefel M, Karatzias T, Ben-Ezra M, Cloitre M, Lueger-Schuster B, Maercker A. The replicability of ICD-11 complex post-traumatic stress disorder symptom networks in adults. Br J Psychiatry. 2019;204(6):361–8.

2. Epskamp S. Package ‘bootnet’: Bootstrap methods for various network estimation routines [Internet]. 2015. Available from: https://cran.r-project.org/web/packages/bootnet/index.html

3. Epskamp S, Cramer AOJ, Waldorp LJ, Schmittmann VD, Borsboom D. Qgraph: Network visualizations of relationships in psychometric data. J Stat Softw. 2012;48(4):1–8.

4. Friedman J, Hastie T, Tibshirani R. Sparse inverse covariance estimation with the graphical lasso. Biostatistics. 2008;9(3):432–41.

5. Foygel R, Drton M. Extended Bayesian information criteria for Gaussian graphical models. In: Advances in Neural Information Processing Systems 23: 24th Annual Conference on Neural Information Processing Systems 2010, NIPS 2010. Vancouver, BC, Canada; 2010. p. 2020–8.

6. Epskamp S, Borsboom D, Fried EI. Estimating psychological networks and their accuracy: A tutorial paper. Behav Res Methods. 2018;50(1):195–212.

7. van Borkulo CD, Boschloo L, Kossakowski JJ, Tio P, Schoevers RA, Borsboom D, et al. Comparing network structures on three aspects: A permutation test. 2017.

8. Yang Z, Algesheimer R, Tessone CJ. A comparative analysis of community detection algorithms on artificial networks. Sci Rep. 2016;6(1):30750.

9. Jones PJ, Ma R, McNally RJ. Bridge centrality: A network approach to understanding comorbidity. Multivariate Behav Res. 2021;56(2):353–67.
